# The Implications of Temperature-Mediated Plasticity in Larval Instar Number for Development within a Marine Invertebrate, the Shrimp *Palaemonetes varians*

**DOI:** 10.1371/journal.pone.0075785

**Published:** 2013-09-19

**Authors:** Andrew Oliphant, Chris Hauton, Sven Thatje

**Affiliations:** Ocean and Earth Science, University of Southampton, National Oceanography Centre Southampton, Southampton, Hampshire, United Kingdom; University of Connecticut, United States of America

## Abstract

Variations in larval instar number are common among arthropods. Here, we assess the implications of temperature-mediated variations in larval instar number for larval development time, larval growth rates, and juvenile dry weight within the palaemonid shrimp, *Palaemonetes varians*. In contrast with previous literature, which focuses on terrestrial arthropods, particularly model and pest species often of laboratory lines, we use wild shrimp, which differ in their life history from previous models. Newly-hatched *P. varians* larvae were first reared at 5, 10, 17, 25, and 30°C to assess their thermal scope for development. Larvae developed at 17, 25, and 30°C. At higher temperatures, larvae developed through fewer larval instars. Two dominant developmental pathways were observed; a short pathway of four instars and a long pathway of five instars. Longer developmental pathways of six to seven instars were rarely observed (mostly at lower temperatures) and consisted of additional instars as ‘repeat’ instars; i.e. little developmental advance over the preceding instar. To assess the implications of temperature-mediated variation in larval instar number, newly-hatched larvae were then reared at 15, 20, and 25°C. Again, the proportion of larvae developing through four instars increased with temperature. At all temperatures, larval development time and juvenile dry weight were greater for larvae developing through five instars. Importantly, because of the increasing proportion of larvae developing through four instars with increasing temperature, larval traits associated with this pathway (reduced development time and juvenile dry weight) became more dominant. As a consequence of increasing growth rate with temperature, and the shift in the proportion of larvae developing through four instars, juvenile dry weight was greatest at intermediate temperatures (20°C). We conclude that at settlement *P. varians* juveniles do not follow the temperature-size rule; this is of importance for life-history ecology in response to environmental change, as well as for aquaculture applications.

## Introduction

Variations in the number of instars during larval development, and the morphology of same stage larvae, within arthropod species is a relatively common plastic trait and is influenced by temperature, salinity, photoperiod, density, sex, food quantity, diet quality, humidity, and pollution (including antibiotics; for reviews see [1], [2]). Within insects, variations in larval instar number are particularly common within orders Lepidoptera and Blattodea (roach spp.) whilst they are lacking within orders Diptera and Hymenoptera (excluding sawflies), suborder Heteroptera (true bugs) and superfamily Papilionoidea (butterflies; for review see [1]). Within crustaceans, they are known for many planktotrophic decapod larvae and appear especially prevalent within euphausiacean, penaeidean, and caridean decapods but are less common within anomurans and brachyurans [2].

Arthropods must periodically moult to grow and develop. Given this inherent biology, larval development is coupled with the moult cycle. Environmental conditions which differently affect the processes of moulting and of growth and development, de-synchronise these processes and drive variations in larval instar number [2], [3]. For some arthropod species, including the tobacco hornworm, *Manduca sexta*, and the forest tent caterpillar, *Malacosoma disstria*, species-specific genetically determined size thresholds (so-called ‘critical weights’) have been shown to initiate pupation and metamorphosis [4]–[7]. Environmental conditions, which slow growth and development, tend to extend larval development until this threshold is achieved and, consequently, result in the addition of larval instars [4], [5]. In contrast, for those arthropod species with a fixed number of larval instars (e.g. the folivorous moth, *Epirrita autumnata*) adult size is strongly dependent on environmental conditions, indicating no size and development threshold for the initiation of metamorphosis [8].

At the inter-specific level within the marine environment, there exists a macro-ecological gradient in larval instar number among marine crustaceans. With increasing latitude, often coupled with decreasing temperature and increasing seasonality and unpredictability of primary production, larval instar number is reduced; see [9], [10]. This is termed abbreviated development and is accompanied by a greater degree of endotrophic development potential. At the intra-specific level, temperature is known to influence larval instar number and has been associated both with additions in larval instar number, and the omitting of larval instars [2], [6], [11], [12] for review see [1]. In some cases, the influence of temperature on variations in larval instar number within marine crustacean species is consistent with the macro-ecological trend in larval instar number at the inter-specific level. For example, within the palaemonid shrimp, *Palaemonetes vulgaris*, larval development proceeds through more larval instars under warmer temperatures [2].

Intra-specific variations in larval instar number are known to influence juvenile and adult body size within arthropods. Such variation in body size may have significant ecological implications. For example, within insects, which do not grow as adults, the accumulation of mass during larval stages affects adult size and several adult traits, including fecundity [13]. Temperature also influences body size among ectotherms. Estimated to occur in >80% of ectotherms (including animals, plants, protozoans, and bacteria), temperature-dependant body size among ectotherms is described by the temperature-size rule (TSR); reared under lower temperatures, organisms grow to a larger body size [14]. However, the influence of temperature-mediated variations in larval instar number on body size and how this plasticity may affect the temperature-size rule are poorly reported.

In an experiment, which sought to identify the thermal limits of larval development within the shrimp, *Palaemonetes varians*, and the influence of temperature on larval development time and juvenile dry weight, we identified temperature-mediated variation in larval instar number. This variation in larval instar number affected both larval development time and juvenile dry weight. We were, therefore, provided the opportunity to assess the influence of temperature-mediated variations in larval instar number on larval development and on body size within *P. varians*.

Working with crustacean larvae is innately difficult because of their small size, the need for tedious individual rearing methods, and high maintenance requirements [15]. Coupled with the fact that the agricultural economic importance of insect pest species has driven research into the larval phase of insects, knowledge of insect larvae is far greater than that of crustaceans [15]. For example, studies assessing the influence of the environment, especially temperature, on development time, growth rate, critical weight, larval instar number and subsequent adult mass have focused on insects, particularly pest and model species such as *Manduca sexta*; e.g. [13], [16], [17]. Laboratory lines of such species, raised under constant conditions and on standard diets for many hundreds of generations, have shown significantly different growth and development rates and less variable development compared with wild type organisms; e.g. [7]. The commercial exploitation of crustacean species within aquaculture now drives a need for understanding better the crustacean larval phase; e.g. [18]. Coupled with the fact that much of what we know about the arthropod larval phase is derived from laboratory lines of model and pest insects, here, we assess the implications of temperature-mediated variations in larval instars number within wild individuals of the caridean shrimp, *Palaemonetes varians*.

## Materials and Methods

### Ethics statement

All experiments followed the legal requirements of the United Kingdom. Experimental manipulations of crustaceans are currently unregulated by the United Kingdom and do not require ethics approval. *Palaemonetes varians* is not a protected or endangered species and no specific permissions were required for field activities.

Experiment 1 (Exp 1; thermal scope for larval development) assessed the temperature range across which successful larval development may take place. This experiment observed temperature-mediated variation in larval instar number, which influenced development time, growth rate and juvenile dry weight (DW). Importantly, given the low fecundity of *Palaemonetes varians*, particularly among the individuals used, coupled with the high number of temperature treatments done, larvae from different females were not divided between all temperatures. Therefore, this experimental design did not account for maternal effects or heritable differences between larvae from different females. This prevented a detailed assessment of the influence of temperature-mediated variation in larval instar number on larval development. Consequently, we carried out Experiment 2 (Exp 2; effects of developmental plasticity) to assess the effects of temperature-mediated variation in larval instar number on larval development time, larval growth rate, and juvenile DW.

For both experiments, larvae used were hatched from wild collected ovigerous *P. varians*. For Experiment 1, 15 ovigerous *P. varians* were collected between June and July, 2011. The field water temperature at the time of collection was 17°C. For Experiment 2, 30 ovigerous *P. varians* were collected between May and June, 2012. The field water temperature was 15°C at the time of collection. All collections and maintenance of shrimp followed the same protocol, detailed below.

### Collections and maintenance

Ovigerous *Palaemonetes varians* were collected by hand-netting from ditches on Lymington salt-marsh (Hampshire, UK) and transferred (within one hour) to the National Oceanography Centre Southampton research aquarium (Hampshire, UK). Here, the embryonic development of broods was assessed and staged according to Müller et al. [19]. Ovigerous *P. varians* with stage VII (final post-nauplius) and VIII (pre-hatching embryo) embryos were selected (after [19]). These ovigerous *P. varians* (with stage VII and VIII embryos) were isolated in 1 litre plastic beakers filled with 850 ml of 17°C (Exp. 1) or 15°C (Exp. 2; field temperatures at the times of collections), 32 salinity, 1 µm-filtered seawater and transferred to temperature-controlled incubators set at 17°C (Exp. 1) or 15°C (Exp. 2) and 12∶12 (L∶D). During the period between collection and larval hatching, ovigerous females were maintained at 17°C (Exp. 1) or 15°C (Exp. 2), 32 salinity, 12∶12 (L∶D) and fed Tetra Goldfish flakes three times per week, to excess. Water changes (>70%) were done every second day. Ovigerous *P. varians* were checked daily (ante meridiem; am) for hatched larvae.

### Larval maintenance

#### Experiment 1 (thermal scope for larval development)

On hatching, actively swimming larvae were separated from 14 ovigerous *P. varians* (using a pipette), haphazardly selected and isolated individually in 100 ml plastic beakers filled with 80 ml of 17°C, 32 salinity, 1 µm- and UV-filtered seawater. Larvae were then transferred to constant temperatures of 5, 10, 17, 25, and 30°C and 12∶12 (L∶D); *n = *96 larvae from three to four females per treatment. This temperature range reflects temperatures naturally experienced by adult populations and the range of adult thermal tolerance [20]–[22]. Water changes (100%) were done daily at 25 and 30°C and every second day at 5, 10, and 17°C. Zoea 1 is facultative lecithotrophic (Oliphant, unpublished data), allowing it to follow an export strategy to estuarine and coastal waters where conditions for larval development may be more favourable ([9] and references therein); consequently, in all treatments zoea 1 larvae were not fed. On moulting to zoea 2, larvae were fed freshly-hatched *Artemia* sp. nauplii (Hobby Artemia) to excess. Larval mortality and development, assessed by morphological changes and moulting (following [3]), were monitored daily (am). On moulting to the juvenile stage, animals were blotted on tissue paper, transferred individually to pre-weighed tin capsules and frozen at −80°C. These samples were later freeze-dried (for 24 hours) and weighed for dry weight (DW, μg).

#### Experiment 2 (effects of developmental plasticity)

Larval maintenance followed the same protocol as above with the following exceptions. Larvae, hatched from 27 ovigerous *P. varians*, were reared at 15, 20, and 25°C; this temperature range falls within that of viable larval development and, given the results of Experiment 1, was considered the most interesting in terms of temperature-mediated variation in larval instar number. Twelve larvae from each of the 27 ovigerous *P. varians* were placed at 15, 20, and 25°C (a total of 324 larvae per temperature), thus this experimental design accounted for maternal effects and heritable differences between larvae from different females. Water changes (100%) were done daily at 25°C and every second day at 15 and 20°C. On moulting to zoea 2, larvae were fed freshly hatched *Artemia* sp. nauplii (ZM systems, decapsulated brineshrimp cysts) to excess.

### Nomenclature of larval stages

Fincham described five zoeal stages for the larval development of *Palaemonetes varians* [3]. Here, Fincham's [3] morphological descriptions were followed to differentiate progressive larval stages; however, terminology for the naming of larval stages differed. The first two larval stages were assigned zoea 1 and zoea 2 (following [3]). The third, fourth, and fifth larval stages were assigned decapodid 1, decapodid 2, and decapodid 3 (following [9], [23]). Fincham [3] reported considerable morphological variation between same stage larvae. Here, morphological variation between same stage larvae was also observed. Rarely, moulting after decapodid 2 occurred with little advance in morphological development – so called ‘repeat’ moults/instars [3], [24], [25]. In Experiment 1, larvae undergoing ‘repeat’ instars developed through five to seven larval instars before metamorphosis. Although larval morphology advanced slightly with ‘repeat’ instars, morphological development was subtle between subsequent larval instars. Studies have identified and named intermediate larval instars of consistent morphology [11], [26]. Here, the number and morphology of ‘repeat’ instars was inconsistent and the naming of intermediate larval instars of consistent morphology was not possible. Instead ‘repeat’ instars are reported here ubiquitously as decapodid’ (D’ in figures). Within a single development pathway a decapodid’ instar was considered to be more morphologically advanced than previous instars; however, decapodid’ instars are not morphologically consistent between larvae with differing moult histories (i.e. in different development pathways). In summary, decapodid 1, 2, and 3 reflect consistent morphological forms (with some morphological variability). Decapodid’ does not reflect consistent morphological forms, but within the same developmental pathway can be considered to advance development.

### Statistical analysis

The effects of development temperature, larval instar number, and the interaction between the two on larval development time, larval growth rate, and juvenile DW were analysed by general linear model (GLM) ANOVA; *post hoc*, multiple comparison of factors, development temperature (°C) and larval instar number (4 vs. 5 instars), were carried out using the Sidak method. Given the low numbers of larvae, which developed through 6 instars (8 of 972) analysis was done on 4 and 5 instars only. All statistical analysis was carried out using Minitab 16 statistical software and in accordance with Sokal and Rohlf [27]. Significance was accepted at *P*<0.05, unless stated otherwise.

## Results

### Experiment 1 (thermal scope for larval development)

At 5°C, all larvae failed to moult to zoea 2 and survived in zoea 1 for an average of 11.8±6.1 days (max. 25 days, min. 3 days). At 10°C, two larvae developed to juvenile (through five instars after 59 and 66 days; respectively); all other larvae died after an average of 55.8±30.1 days (max. 107 days, min. 1 day). Larval development at 10°C proceeded normally until decapodid 2. After decapodid 2, very little to no development was evident between moults and so these were considered ‘repeat’ instars (decapodid’). The instar duration (and standard errors) of these ‘repeat’ instars (decapodid’) was greater than for zoea 1, zoea 2, decapodid 1, and decapodid 2 (Table 1). Similarly, survivorship during ‘repeat’ moults (decapodid’) was lower than during zoea 1, zoea 2, decapodid 1, and decapodid 2 moults (Table 1).

**Table 1:** *Palaemonetes varians* instar duration (presented as means±standard errors) and survival within instars and cumulative survival from hatching at 10°C. Instars are indicated, initial *n = *96.

		Z1	Z2	D1	D2	D'	D'	D'	D'
Instar	days	6.4	11.4	10.8	11.1	13.5	11.8	14.2	14.3
duration	(±S.E.)	(±0.2)	(±0.2)	(±0.1)	(±0.2)	(±0.7)	(±0.5)	(±1.6)	(±3.2)
									
Survival	%	90.6	88.5	93.5	93.1	79.1	62.3	39.4	23.1
	**cum.%**	**90.6**	**80.2**	**75.0**	**69.8**	**55.2**	**34.4**	**13.5**	**3.1**

Dashed line indicates the point at which two individuals (2.1%) metamorphosed to juvenile. Z indicate zoeal stages, D indicate decapodid stages.

**Figure 1:**
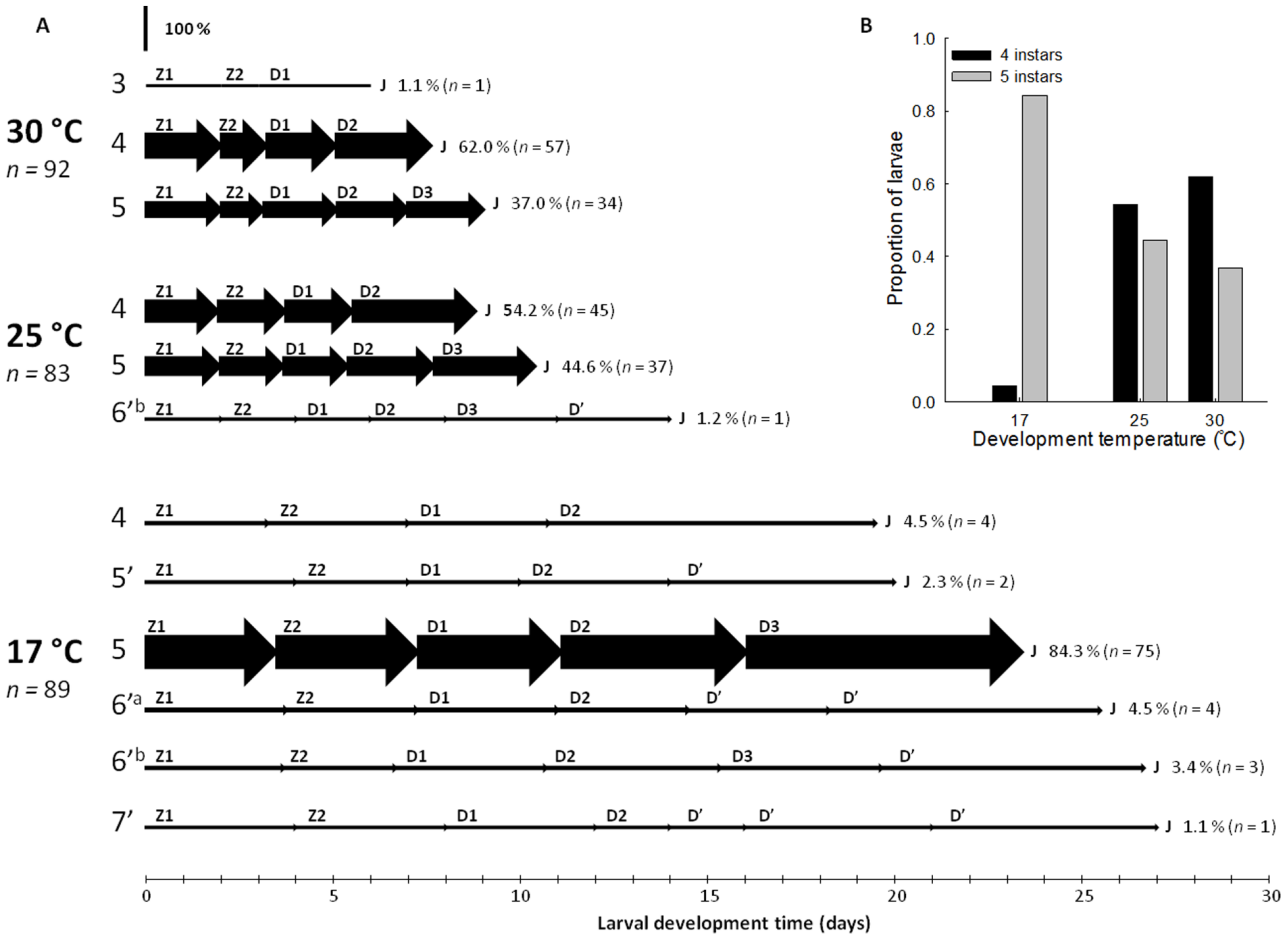
Temperature-mediated developmental plasticity within *Palaemonetes varians* larvae. (A) Diagram of *Palaemonetes varians* larval development pathways observed during Experiment 1 (thermal scope for larval development) reared at three temperatures (17, 25, 30°C). Arrow height = per cent of larvae developing through a pathway (also indicated at the end of the development pathway), arrow length = instar duration (days). Development pathways and instars are indicated. (B) Proportions of larvae developing through four and five instars at 17, 25, and 30°C.

Successful larval development occurred at 17°C (92.7% metamorphosed to juvenile), 25°C (86.5%), and 30°C (95.8%). Larval development proceeded through varying development pathways, consisting of different numbers of larval instars (Figure 1.A). Two development pathways were numerically dominant, a five instar pathway (“5” in Figure 1.A; as described by Fincham [3]) and a four instar pathway (“4” in Figure 1.A); larvae metamorphosed to juveniles directly from decapodid 2, omitting decapodid 3. Alternative development pathways, consisting of five to seven larval instars predominantly occurred at 17°C. The 5′ development pathway proceeded via a single ‘repeat’ instar (decapodid’) after decapodid 2; 6′^a^ consisted of two repeat instars after decapodid 2; 6′^b^ consisted of a single ‘repeat’ instar after decapodid 3; and 7′ consisted of three ‘repeat’ instars after decapodid 2 (Figure 1.A). Such development was rare, occurring in 11.2% of larvae at 17°C, 1.2% at 25°C, and was absent at 30°C. Interestingly, at 30°C a single larva metamorphosed to juvenile directly from decapodid 1, omitting decapodids 2 and 3. Temperature appeared to influence larval development pathway; larvae increasingly developed through four instars at higher temperatures. With increasing temperature, the proportion of larvae developing through five instars decreased from 0.84 at 17°C to 0.37 at 30°C. The number of larvae developing through four instars increased with temperature from 0.04 at 17°C to 0.62 at 30°C (Figure 1.B).

### Experiment 2 (effects of developmental plasticity)

#### Developmental plasticity

**Figure 2:**
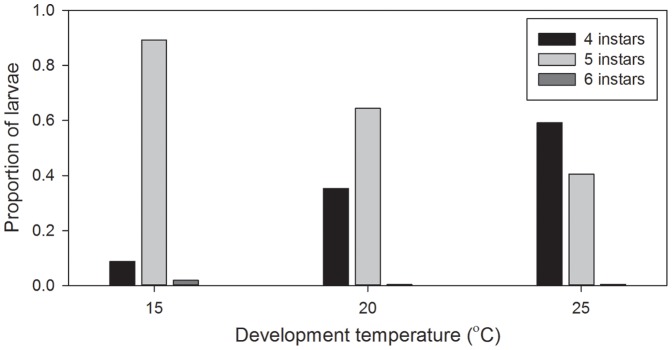
Proportions of *Palaemonetes varians* larvae developing through four, five, and six instars at 15, 20, and 25 °C.

Larval instar number was influenced by temperature, consistent with the results of Experiment 1; larvae increasingly developed through fewer instars with increasing temperature (Figure 2.). The proportion of larvae developing through four instars increased from 0.09 at 15°C to 0.59 at 25°C, whilst the proportion developing through five instars decreased from 0.89 at 15°C to 0.40 at 25°C (Figure 2.). The dominant pathway for larval development was, therefore, five instars at 15°C, five instars at 20°C, and four instars at 25°C. Larval instar number was less variable than in Experiment 1, larvae predominantly develop through four or five instars; <1% of larvae developed through six instars.

#### Development time

**Figure 3:**
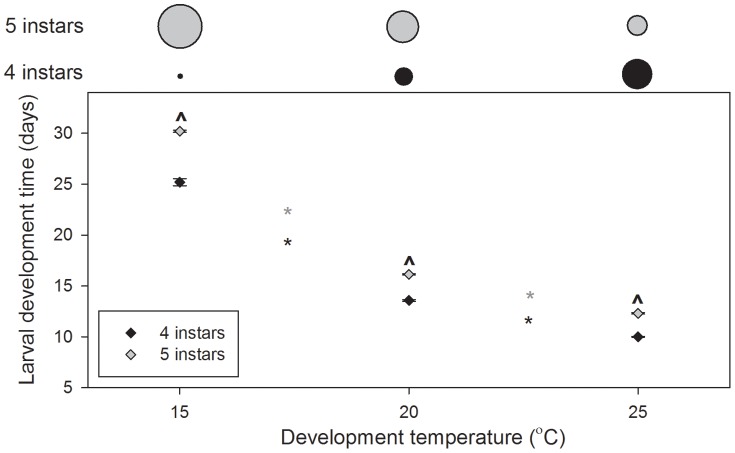
*Palaemonetes varians* larval development times through four and five instars at 15, 20, and 25°C. Data are presented as means±standard errors. Asterisks indicate significant differences between temperatures (*, five instars; *, four instars), ? indicate differences (within temperatures) between larvae developing through four and five instars. Above the plot, circle diameters indicate the proportion of larvae developing through four (black) and five instars (grey) at each temperature.

The effects of development temperature, larval instar number, and the interaction between them, on development time were significant (*P<*0.001 in all cases; Figure 3.). Development time through both four and five instars decreased significantly with increasing temperature (*P<*0.001 in all cases; Figure 3.). Within development temperatures, development time was significantly longer for larvae developing through five instars (*P<*0.001 in all cases; Figure 3.). Development through five instars took 19.9% longer at 15°C, 18.9% longer at 20°C, and 23.1% longer at 25°C. The proportion of larvae developing through this longer development pathway (five instars) decreased with increasing temperature (Figure 3.).

#### Juvenile DW

**Figure 4:**
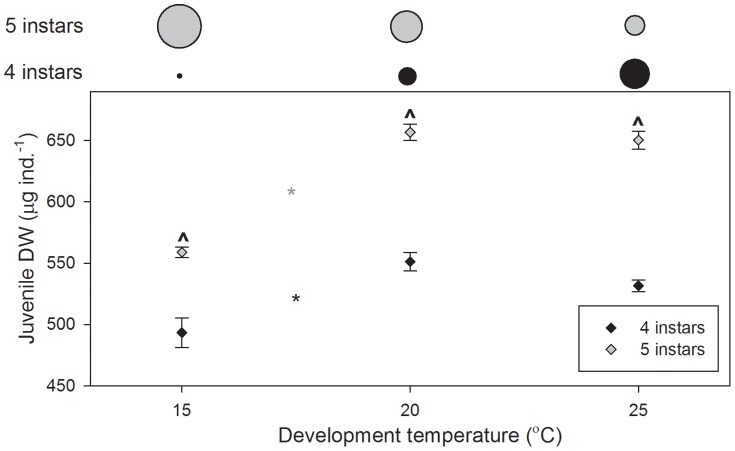
*Palaemonetes varians* juvenile DW after development through four or five larval instars at 15, 20, and 25°C. Data are presented as means±standard errors. Asterisks indicate significant differences between temperatures (*, five instars; *, four instars), ? indicate differences (within temperatures) between larvae developing through four and five instars. Above the plot, circle diameters indicate the proportion of larvae developing through four (black) and five instars (grey) at each temperature.

Juvenile DW was significantly affected by development temperature (*P<*0.001), larval instar number (*P<*0.001) and the interaction between the two (*P = *0.043; Figure 4.). For both four and five instars, juvenile DW increased with increasing temperature between 15 and 20°C, only (*P<*0.0001; Figure 4.). Within development temperatures, juvenile DW was greater for larvae developing through five instars (*P = *0.002 at 15°C, *P<*0.001 at 20 and 25°C; Figure 4.). Juvenile DW after development through five instars was 13.3% greater at 15°C, 19.1% greater at 20°C, and 22.3% greater at 25°C. The proportion of larvae developing to a larger size through this longer development pathway (five instars) decreased with increasing temperature (Figure 4.).

#### Growth rate

**Figure 5:**
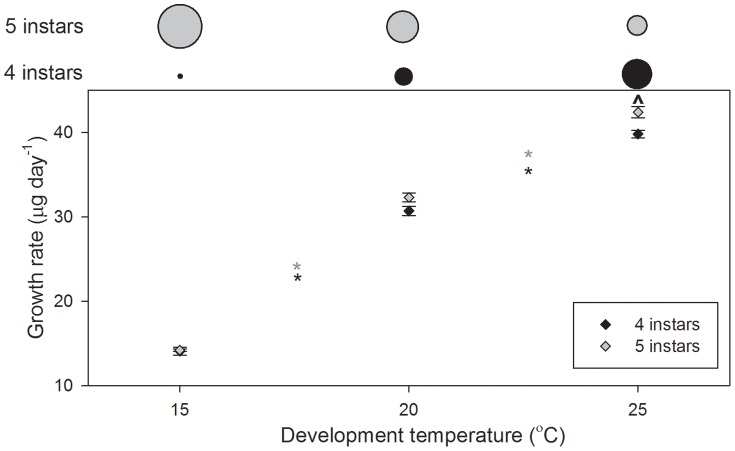
*Palaemonetes varians* growth rates for larvae developing through four and five instars at 15, 20, and 25°C. Data are presented as means±standard errors. Asterisks indicate significant differences between temperatures (*, five instars; *, four instars), ? indicate differences (within temperatures) between larvae developing through four and five instars. Above the plot, circle diameters indicate the proportion of larvae developing through four (black) and five instars (grey) at each temperature.

Growth rates were affected by development temperature (*P<*0.001), larval instar number (*P = *0.006), but not the interaction between them (Figure 5.). Growth rate for both four and five instars increased significantly with increasing temperature (*P<*0.001 in all cases; Figure 5.). Within development temperatures, differences in growth rates between larvae developing through four and five instars were significant at 25°C, only (*P = *0.0013).

## Discussion

Temperature-mediated variation in larval instar number is known among arthropods; additional larval instars have been observed under both higher and lower temperatures [2], [6], [11], [12] for review see [1]. Here, we demonstrate that under conditions of high growth rates (high temperature), *P. varians* larvae tend to omit larval instars whilst under conditions of low growth rates (low temperature) additional larval instars may occur during development. With increasing temperature, greater growth and development rates likely provided the opportunity for earlier metamorphosis; i.e. larvae developed sufficiently by decapodid 2 to moult to juvenile directly, omitting decapodid 3. At lower temperatures, additional instars were observed as ‘repeat’ instars (decapodid’) suggesting that morphogenesis and growth between larval instars was retarded. The predominance of additional larval instars under lower temperatures indicates that these temperatures are suboptimal for larval development within *P. varians*.

Wild larvae from plankton trawls may be represented by fewer larval instars than laboratory reared larvae of the same species; e.g. [3], [24], [25]. Morphological variation and differences in the extent of development and larval size are also known between wild and laboratory reared larvae [28], [29]. Further, the extension of larval development through a series of decapodid moults, each of which advances development little, has been considered a plastic response to a lack of settlement cue [29]. These observations indicate that laboratory experiments may not fully replicate the conditions under which wild larvae develop. Here, we document the effects of temperature on larval development and demonstrate temperature-mediated plasticity in larval instar number. The ability for decapod larvae to cope with sub-optimal conditions through plasticity in instar number is considered advantageous and may, potentially occur in wild larvae where it may have ecological implications.

Among arthropods, variation in larval instar number is a common plastic trait. This trait results from the interaction between the inherent biology of arthropods, which must moult to grow and develop, and the differential effects of the environment on the moult cycle and on growth and development rates [4], [5], [7]. Unlike non-arthropods which grow incrementally, within arthropods variations in larval instar number may give rise to differences in juvenile and adult DW because, although growth and development thresholds may be reached during an instar, growth and development continues until the end of the moult cycle. For example, here we show that for *Palaemonetes varians*, development through four instars is quicker and gives rise to juveniles of reduced DW than development through five instars, across all temperatures. Similarly, within the estuarine crab, *Neohelice (Chasmagnathus) granulata*, larvae may omit an instar during development; larvae, which do omit an instar metamorphose sooner but to juveniles of reduced DW [30], [31]. For the tobacco hornworm, *Manduca sexta*, and the forest tent caterpillar, *Malacosoma disstria*, larval instar number affects development time and mass; those larvae developing through more instars take longer to develop but do so to a larger size [4], [6], [7]. Considering the pervasive nature of variations in larval instar number coupled with the varied life histories among arthropods, moulting, which may give rise to considerable differences in larval development time and juvenile or adult DW, may have significant and diverse ecological implications. For example, within the holometabolous insects, reproductively active adults emerge after pupation. For *M. sexta,* pupal mass (which is influenced by larval instar number) is positively correlated with the number of mature eggs in the ovaries after eclosion [7], [13]. For settling crustaceans, however, juveniles are not reproductively active but continue to grow into adulthood.

### Insight into competence thresholds for settlement

**Figure 6:**
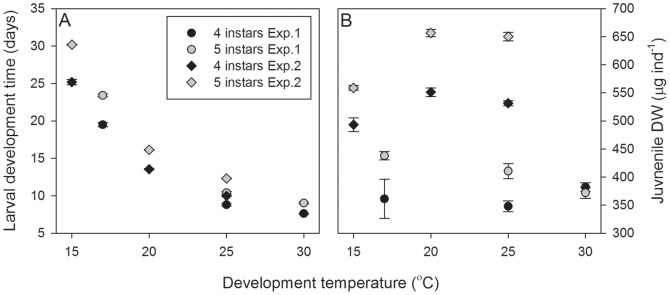
Comparison of larval development time and juvenile dry weight between Experiments 1 and 2. (A) *Palaemonetes varians* larval development times for larvae developing through four and five instars in Experiment 1 (at 17, 25, 30°C) and in Experiment 2 (at 15, 20, 25°C). Data are presented as means±standard errors. (B) *Palaemonetes varians* juvenile DW after development through four or five larval instars in Experiment 1 (at 17, 25, 30°C) and in Experiment 2 (at 15, 20, 25°C). Data are presented as means±standard errors.

Suboptimal environmental and ecological conditions tend to increase larval instar number and reduce morphogenesis between larval instars in crustaceans with high developmental plasticity (e.g. euphausids and carideans; [15]). Diet is known to influence variations in larval instar number [1], [2]. Consequently, the use of different suppliers of *Artemia* sp. within Experiments 1 and 2 (Hobby vs. ZM systems, respectively) may explain differences in the frequency of additional instars, juvenile DW (Figure 6.B) and larval growth rates (data not shown) between the experiments. Similarly, larvae were derived from different females and in different years. Maternal effects may therefore have influenced differences between experiments. Nevertheless, the effects of temperature on larval instar number were found to be consistent between experiments.

The potential effects of diet or uncontrolled maternal effects on the current study are reflected in a comparison of juvenile DWs between Experiments 1 and 2 (Figure 6.A and 6.B). Whilst larval development rates were consistent between both experiments the juvenile DWs were not. Coupled with temperature-mediated variations in larval instar numbers, these data suggest that *P. varians* do not develop to a threshold size but continuously develop from larvae to juveniles. Environmental conditions which perturb the rate of development result in variations in larval instar number. We do not suggest that there is not a minimum size a larva may be, but that there is no size threshold for the end of larval development. This may be true of decapod larvae in general.

### Macro-ecological perspective

Temperature-mediated variations in larval instar number observed here for *Palaemonetes varians* are inconsistent with those made for *Palaemonetes vulgaris* and may be associated with the relative amounts of per offspring investment and associated endotrophic development potential within these species. *Palaemonetes vulgaris* is fully planktotrophic and unable to develop to its second larval stage (zoea 2) without feeding [32]. *Palaemonetes varians* is facultative lecithotrophic during its first larval stage (zoea 1; Oliphant unpublished) and can develop to its third and sometimes fourth larval stages (decapodid 1 and 2, respectively) without feeding [33]. The higher internal reserves present within *P. varians* larvae may allow for more rapid development at higher temperatures. For example, under starved conditions, *P. varians* larvae develop to more advanced larval stages under warmer conditions [33]. Results were also inconsistent with the macro-ecological trend in larval instar number at the inter-specific level. At the inter-specific level, species with abbreviated development have high per offspring investment and high endotrophic development potential. Within *P. varians*, Oliphant and Thatje [33] showed that under the same conditions, larvae from broods of higher hatchling energy content developed through fewer larval instars. This suggests that the inconsistency between observations at the intra-specific level and those at the inter-specific level are linked to inter-specific differences in per offspring investment.

### Palaemonetes varians and the temperature-size rule

Studies of larval development within insects have provided better understanding of the temperature-size rule. For example, within *Manduca sexta*, larval body size (which follows the temperature-size rule) was found to be a function of the “interval to cessation of growth (ICG)”- the period between attaining the critical weight for the initiation of pupation (which is thermally invariable) and the initiation of pupation. The ICG decreased with temperature, providing a shorter time for mass accumulation and resulting in smaller larvae at higher temperatures [13], [34]. In contrast, Ghosh et al. [35] identified genetically controlled shifts in the critical weight for pupation with temperature within *Drosophila*. Importantly, such studies use laboratory lines of model species, which may differ significantly from wild types of the same species. For example, under the same constant conditions, wild type *Manduca sexta* exhibit temperature-mediated variation in larval instar number whilst laboratory types do not [7].

Our data suggest that *P. varians* do not develop to a size threshold but develop continuously to juveniles. Even so, temperature influences juvenile size. Variations in larval instar number, coupled with higher growth rates at higher temperature, gave rise to the greatest juvenile DWs at intermediate temperatures (20°C). Consequently, temperature-mediated variation in larval instar number and its interaction with temperature-mediated growth rates is important in determining juvenile DW. Larval instar number is therefore of high importance in understanding the effects of temperature on decapod larval development. Interestingly, this is rarely considered in literature assessing the implications of temperature for larval growth, which is highly important to the aquaculture industry; e.g. [36], [37], [38]. For *Artemia franciscana*, Forster and Hirst [39] found that for early ontogenetic stages, individuals were larger at higher temperatures and that the typical temperature-size rule became established only in later ontogenetic stages. This was thought to result from high temperature dependence in growth rate in early ontogenetic stages, which reduced through later stages. *Palaemonetes varians* may follow a similar pattern, with juvenile size being influenced by temperature-mediated variation in larval instar number and its interaction with temperature-mediated growth rates.

### Ecological implications

Size at metamorphosis has important fitness consequences as larger individuals are considered fitter [40], [41]. For settling decapods, a large size may increase starvation resistance, increase predation success, and reduce cannibalism by fellow new-recruits [31]. Accordingly, *P. varians* juveniles whom developed through five instars may be considered fitter at all temperatures. The juvenile environment may have important consequences for the fitness implications of size at metamorphosis. For example, under fed conditions, short pathway *N. granulata* juveniles grew faster than long pathway juveniles, indicating that any disadvantage of small size at metamorphosis may be reduced through subsequent instars [31]. Similarly, Sandifer and Smith [42] found no growth or development advantage for early (with fewer larval instars), compared to late (with more larval instars) metamorphosing juvenile *Macrobrachium rosenbergii*. Later metamorphosing juveniles were smaller (in terms of carapace length) than earlier metamorphosing juveniles; however, after three weeks, no difference was evident [42]. These findings suggest that the influence of developmental plasticity (and larval experience) on initial juvenile fitness (and possibly later life stages) is likely to depend on habitat characteristics; advantages associated with larger juveniles may be greater under suboptimal conditions [31]. For *P. varians*, conditions which drive high growth rates during the larval phase promote development through four instars and settlement at a reduced DW. An important factor influencing the effects of the larval experience on later life stages appears to be the relationship between conditions during the larval phase and those after settlement. Poor conditions during development lead to development through more larval stages, giving rise to large juveniles. Good conditions during development lead to development through fewer larval instars, giving rise to smaller juveniles. The literature suggests that larger individuals are fitter if conditions after settlement are poor. If conditions after settlement are good, there is no advantage of large size. Consequently, if conditions during the larval period do not reflect those after settlement, variation in larval instar number may be disadvantageous.

For dispersive propagules such as the planktonic larvae of aquatic arthropods, additional larval instars and delayed recruitment (resulting from slower epigenesis under sub-optimal conditions) or the omission of larval instars and earlier recruitment (resulting from faster epigenesis under more favourable conditions) will affect larval transport; an indirect effect which could enhance or reduce dispersion and affect the chances of finding suitable conditions for growth and settlement [15]. Strong dispersion promotes gene flow and is associated with colonisation/re-colonisation of habitats and low extinction rates; however, dispersal may be disadvantageous in the short-term (for review see [43]). For example, when local conditions are optimum (either temporally or spatially) reduced development time to settlement may enhance local recruitment in the favourable habitat [15]. Further, a short planktonic phase may also reduce the risks of mortality via predation in the plankton. For *P. varians*, when conditions are favourable and larval development is rapid, local recruitment will be maximised. Conversely, if conditions are unfavourable, development will be slow, prolonging the larval period and increasing dispersal; possibly to more favourable environments.
