# Correction: The Implications of Temperature-Mediated Plasticity in Larval Instar Number for Development within a Marine Invertebrate, the Shrimp *Palaemonetes varians*

**DOI:** 10.1371/annotation/52e4f765-b1a6-4286-8d44-79e2bf0ca8aa

**Published:** 2014-01-13

**Authors:** Andrew Oliphant, Chris Hauton, Sven Thatje

An error occurred in Table 1 while this article was being prepared for publication. The dashed line referenced in the Table 1 legend is missing and the alternate shading of the rows make the table difficult to read. Please see the corrected version of Table 1 here: http://www.plosone.org/corrections/pone.007578.001.cn.tif

Errors also occurred in the Figure 3, 4, and 5 legends. For all three figure legends, "? indicate differences (within temperatures)" should instead read "^ indicate differences (within temperatures)." Additionally, for all three figure legends, in the statement "(*, five instars; *, four instars)", the first asterisk representing five instars should be grey to correspond to the grey asterisks in these figures.

